# Supplementary material for: Iterative improvement in the automatic modular design of robot swarms
Source: PeerJ Comput Sci. 2020 Dec 7;6:e322. doi: 10.7717/peerj-cs.322 (PMC7924708; doi:10.7717/peerj-cs.322)
Supplement: Supplemental Information 3 [file peerj-cs-06-322-s003.zip › argos3/doc/api/standalone/a00549_source.html]

ARGoS: plugins/robots/mini-quadrotor/control\_interface/ci\_miniquadrotor\_trajectory\_actuator.h Source File


- Main Page
- Related Pages
- Namespaces
- Classes
- Files

- File List
- File Members

# plugins/robots/mini-quadrotor/control\_interface/ci\_miniquadrotor\_trajectory\_actuator.h

Go to the documentation of this file.

```
00001 
00007 #ifndef MINIQUADROTOR_TRAJECTORY_ACTUATOR_H
00008 #define MINIQUADROTOR_TRAJECTORY_ACTUATOR_H
00009 
00010 namespace argos {
00011    class CCI_MiniQuadrotorTrajectoryActuator;
00012 }
00013 
00014 #include <argos3/core/control_interface/ci_actuator.h>
00015 #include <argos3/core/utility/math/vector3.h>
00016 
00017 namespace argos {
00018 
00019    class CCI_MiniQuadrotorTrajectoryActuator : public CCI_Actuator {
00020 
00021    public:
00022 
00023       struct SWaypoint {
00024          CVector3 Position;
00025          CRadians Yaw;
00026 
00027          SWaypoint();
00028          SWaypoint(const CVector3& c_position,
00029                    const CRadians& c_yaw);
00030       };
00031 
00032    public:
00033 
00034       virtual ~CCI_MiniQuadrotorTrajectoryActuator() {}
00035 
00036       virtual void SetWaypoint(SWaypoint& s_waypoint) = 0;
00037 
00038 #ifdef ARGOS_WITH_LUA
00039       virtual void CreateLuaState(lua_State* pt_lua_state);
00040 #endif
00041 
00042    };
00043 
00044 }
00045 
00046 #endif
```

---

Generated on 10 Jul 2018 for ARGoS by 
 1.6.1
